# B Lineage Cells in ANCA-Associated Vasculitis

**DOI:** 10.3390/ijms23010387

**Published:** 2021-12-30

**Authors:** Ana Merino-Vico, Jan Piet van Hamburg, Sander W. Tas

**Affiliations:** 1 Department of Rheumatology and Clinical Immunology, Amsterdam Rheumatology and Immunology Center, Amsterdam University Medical Centers, University of Amsterdam, Meibergdreef 9, 1105 AZ Amsterdam, The Netherlands; a.merinovico@amsterdamumc.nl (A.M.-V.); j.p.vanhamburg@amsterdamumc.nl (J.P.v.H.); 2 Department of Experimental Immunology, Amsterdam University Medical Centers, University of Amsterdam, Meibergdreef 9, 1105 AZ Amsterdam, The Netherlands

**Keywords:** B cells, ANCA-associated vasculitis, ANCA, plasma cells, signalling, autoimmunity

## Abstract

Anti-neutrophil cytoplasmic antibody (ANCA)-associated vasculitis (AAV) is a systemic autoimmune disease that affects small sized blood vessels and can lead to serious complications in the lungs and kidneys. The prominent presence of ANCA autoantibodies in this disease implicates B cells in its pathogenesis, as these are the precursors of the ANCA-producing plasma cells (PCs). Further evidence supporting the potential role of B lineage cells in vasculitis are the increased B cell cytokine levels and the dysregulated B cell populations in patients. Confirmation of the contribution of B cells to pathology arose from the beneficial effect of anti-CD20 therapy (i.e., rituximab) in AAV patients. These anti-CD20 antibodies deplete circulating B cells, which results in amelioration of disease. However, not all patients respond completely, and this treatment does not target PCs, which can maintain ANCA production. Hence, it is important to develop more specific therapies for AAV patients. Intracellular signalling pathways may be potential therapeutic targets as they can show (disease-specific) alterations in certain B lineage cells, including pathogenic B cells, and contribute to differentiation and survival of PCs. Preliminary data on the inhibition of certain signalling molecules downstream of receptors specific for B lineage cells show promising therapeutic effects. In this narrative review, B cell specific receptors and their downstream signalling molecules that may contribute to pathology in AAV are discussed, including the potential to therapeutically target these pathways.

## 1. Introduction

Anti-neutrophil cytoplasmic antibody (ANCA)-associated vasculitis (AAV) is a systemic autoimmune disease characterised by the presence of ANCA autoantibodies in the serum of patients. Autoreactive B cells secrete ANCAs which in their turn initiate inflammatory signalling cascades, eventually leading to endothelium damage of small-size blood vessels of mainly renal glomeruli and the respiratory tract [1]. Autoantibodies such as ANCAs are one of the major hallmarks of autoimmunity as they may target self-antigens and promote inflammation. Their pathogenic role has not only been described in AAV, but also in various other autoimmune diseases, including systemic lupus erythematosus (SLE) and rheumatoid arthritis (RA) where anti-double stranded DNA antibodies or anti-citrullinated protein antibodies (ACPAs) are present both in serum and inflamed tissues of patients, respectively. The importance of autoantibodies in autoimmunity underscores the crucial role of the cells involved in their generation and production: B cells and plasma cells (PCs).

Besides autoantibody production, B cells have been found to contribute to disease pathogenesis in AAV and other autoimmune diseases through antibody-independent functions, including cytokine secretion and antigen presentation to T cells [2,3]. Direct confirmation of the role of B cells in AAV and other autoimmune diseases emerged from the beneficial effects of B cell depletion therapy using the anti-CD20 monoclonal antibody rituximab (RTX) [4,5,6,7]. However, the variable efficacy in some patients and the inability of RTX to target autoantibody-producing PCs, makes it necessary to improve existing or to develop new B lineage targeted therapies [8]. In the search for potential targets, it is essential to identify the key features of B lineage cells in AAV—including specific B lineage cell populations, surface receptors, downstream signalling cascades, and effector molecules—as better understanding of these features may contribute to defining specific therapeutic targets that may only affect activated and/or autoreactive cells. This review aims to recapitulate the latest findings on B lineage cells and their critical receptors and downstream molecules in AAV.

## 2. ANCA-Associated Vasculitis (AAV): Brief Background on Clinical Picture and Current Treatment Strategies

AAV encompasses three major types of vasculitides that have different clinical characteristics, namely granulomatosis with polyangiitis (GPA, previously referred to as Wegener’s granulomatosis), microscopic polyangiitis (MPA), and eosinophilic granulomatosis with polyangiitis (EGPA) [9]. AAV vasculitides are characterized by pauci-immune features (i.e., limited/absence of ANCA-immune complexes in renal glomeruli) [10,11] and by the presence of circulating ANCAs in the serum of patients, which may be specific for different neutrophil antigens, most commonly myeloperoxidase (MPO) and proteinase 3 (PR3). However, there are also minor antigens and even patients who are serologically ANCA-negative (approximately 4–14%) [12,13,14,15]. Interestingly, most studies indicate that ANCA-negative patients are more frequently diagnosed with GPA than with MPA [13,14,15]. GPA is identified by the presence of granulomatous inflammation and necrosis, mainly in the small vessels of the respiratory tract and/or kidneys. Interestingly, GPA-AAV patients mostly are PR3-ANCA positive (85%) [16], while a small percentage is MPO-ANCA positive [1]. MPA is characterized by necrotizing vasculitis, often with renal involvement, and most patients are MPO-ANCA positive (60%). Clinical characteristics of EGPA are largely similar to that of GPA, but in addition patients present with high eosinophil levels and only 50% of patients has circulating ANCAs, the majority being MPO-ANCA positive whereas PR3-ANCA is rare in EGPA [1]. Recent studies that aimed to find a more robust classification system of AAV patients revealed a more significant association of genotype, response to induction and maintenance of remission, and risk of relapse with ANCA-specificity (i.e., PR3 or MPO) [17,18] than with clinical diagnosis [19]. Hence, a gradual transition towards a classification based on ANCA-specificity may lead to a better diagnosis and more homogenous cohorts in clinical trials in the near future [19,20].

The aetiology of AAV is poorly understood and complex, involving genetics and epigenetics, environmental factors such as drugs or (previous) infections, and ANCAs [1]. The pathogenicity of ANCAs relies on their ability to activate neutrophils, which subsequently release pro-inflammatory cytokines, reactive oxygen species (ROS) during the respiratory burst, and lytic enzymes, leading to endothelial cell damage in glomeruli and the respiratory tract. ANCAs can trigger a pathogenic inflammatory response, implicating different immune cell types, to which the alternative pathway of the complement system also contributes, as revealed by elevated serum levels of C5a in AAV patients [21]. Of note, the pathogenicity of MPO-ANCAs is more firmly established than that of PR3-ANCAs [22].

Current AAV treatment strategies aim to induce disease remission and often include cyclophosphamide or RTX [23,24,25], followed by maintenance therapy using immunosuppressive agents such as azathioprine, methotrexate, mycophenolate, or more recently also RTX [26,27,28]. Cyclophosphamide, an alkylating agent, was the gold standard for immunosuppression in cancer for a long time and its effects on B cells, in particular the inhibition of (auto)antibody production in autoimmunity was recognized [27,29], resulting in the incorporation of cyclophosphamide in remission-induction treatment strategies in severe manifestations of various autoimmune diseases. However, the severe adverse events of this drug [30,31] lead researchers to search for alternative therapeutic agents for induction of remission in AAV. RTX depletes CD20-expressing B cells via distinct mechanisms of action, including antibody-dependent cellular cytotoxicity (ADCC), complement-dependent cytotoxicity, and induction of apoptosis [3]. The potential of RTX in the induction of remission in AAV was investigated in several clinical trials, including the pioneering RAVE and RITUXVAS studies, which demonstrated similar clinical effects of RTX compared to cyclophosphamide (both combined with glucocorticoids) in severe AAV, with comparable or even less adverse events [23,24]. Furthermore, RTX seemed to be superior in patients with relapsing disease [23]. One study focusing on the Wegener’s Granulomatosis Etanercept and RAVE trials compared the ANCA-negative subgroup of patients diagnosed with GPA versus the PR3-ANCA positive GPA patients, and concluded that the ANCA-negative patients had lower Birmingham Vasculitis Activity Scores at baseline (mainly due to less renal involvement), but a similar rate of relapse during a 2-year follow-up [14]. In 2015, RTX was tested in 14 patients with AAV presenting with severe renal disease in combination with glucocorticoids, and all patients—both ANCA-positive and ANCA-negative—exhibited high rates of remission [13]. Consequently, ANCA-negative patients can also benefit from remission-induction using RTX.

Following induction therapy, methotrexate and azathioprine have similar outcomes and safety profiles as maintenance therapy agents [32], and when compared with mycophenolate, the latter was found to be superior to azathioprine at suppressing cytokine production by B cells in a small patient cohort [33]. Of note, RTX has been suggested to be a better alternative than azathioprine for maintenance of remission [28], which was confirmed in a more recent study [34]. However, both studies emphasize the need to evaluate the long-term effects of RTX as maintenance therapy. Despite its clear clinical benefits in many patients, RTX only targets peripheral circulating B cells, which allows long-lived plasma cells to remain a source of ANCA production [23]. These results illustrate the unmet medical need in AAV and warrants the search for new or improved therapeutic approaches for AAV patients.

## 3. B Lineage Cells in AAV: Differentiation, Prevalence, and Function

B cells originate from hematopoietic stem cells in the bone marrow (BM) where they undergo several differentiation steps to give rise to mature B cells. During this process, the B cell receptor (BCR) undergoes genetic rearrangements creating a diverse repertoire [3]. Self-antigens are presented in the BM, and a subsequent rigorous selection mechanism (central tolerance) ensures that autoreactive B cells do not egress the BM. In this manner, only B cells expressing BCRs with no or low avidity for self-antigens are positively selected and enter the circulation. Of note, during B cell development, all populations express CD19 and CD20 (coreceptors of the BCR), until they reach the PC stage which is characterized by a downregulation of CD20.

**Figure 1:**
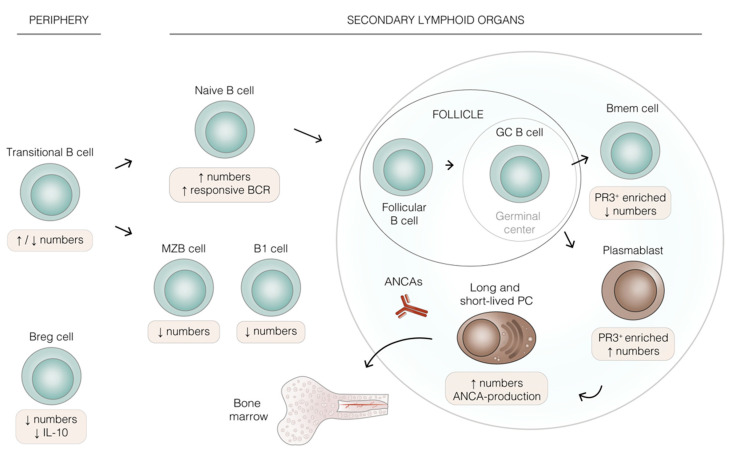
B cell lineage development and altered B cell populations in ANCA-associated vasculitis (AAV). Schematic representation of human B lineage cell differentiation. Beige boxes highlight the effects of AAV on the indicated cell populations. The extra-follicular pathway of B cell differentiation is not shown for simplicity. Transitional B cells migrate into secondary lymphoid organs, where they differentiate into innate-like B1, marginal zone B (MZB) cells, or naive B cells. Naive B cells enter lymph nodes and become follicular B cells, and it is within the follicle that a T-cell dependent response triggers the formation of germinal centers (GCs). During the GC reaction, the GC B cell may generate memory B cells (Bmem), plasmablasts, or plasma cells (PCs), which reduce the pathogenic anti-neutrophil cytoplasmic antibodies (ANCAs). PCs may be short- or long-lived and home to the bone marrow, whereas Bmem egress into the periphery. B regulatory cells (Breg) may arise from almost all B cell lineage populations. All the aforementioned B cell populations have been found to be dysregulated in AAV, being increased or decreased, as shown by the arrows in the beige boxes. Abbreviations: PR3 (proteinase 3), BCR (B cell receptor).

Transitional B cells (CD24^+^CD38^+^) are immature BM emigrants that express IgM on their surface (Figure 1). These cells can be found in circulation upon their homing to the spleen [35,36], the main secondary lymphoid organ where they are able to complete their maturation in three steps (T1, T2, and T3), defined by IgD upregulation and IgM downregulation [37]. Studies regarding potential alterations in the number of transitional B cells in AAV are not conclusive and a better understanding is needed. Some studies report increased transitional B cells in GPA patients compared to controls [33,38], whereas an earlier study found a reduction in transitional B cells in active disease compared to patients in remission and HCs [39]. Transitional B cells give rise to the B lineage subtypes B1, marginal zone B cells (MZB), or follicular B cells [35].

B1 cells (CD27^+^CD43^+^CD70^−^) [40] are innate-like cells which produce IgM and populate mainly the gut and lung epithelia. MZB (IgD^+^CD27^+^IgM^hi^), on the other hand, mostly reside in the spleen and respond to mostly T cell-independent stimuli. Upon T cell-independent antigen encounter, MZB cells may enter extra-follicular foci and differentiate into short-lived plasma cells that can produce antibodies of lower affinity and broad specificity (IgM) [26]. Both B1 and MZB cells are involved in innate and adaptive immunity [3], and they were found to be reduced in circulation in patients with active AAV [41]. This study also identified MZB cells as the main IgM producers of all circulating B cells and, together with B1 cells, they secreted the anti-inflammatory cytokine IL-10 at the same extent as healthy controls (HCs), suggesting a potential role for these cells in maintaining immune homeostasis in AAV.

Follicular B cells are mature naive B cells found in specialized structures known as follicles in secondary lymphoid organs—i.e., the spleen itself, lymph nodes, and Peyer’s patches. Follicular B cells (IgD^+^CD27^−^CXCR5^+^) unlike MZB cells, are activated in a T cell-dependent manner within follicular areas. During this process, cognate antigen ligation and subsequent T cell help allow germinal center (GC) formation and the GC reaction to occur [26]. This orchestrated process promotes extensive B cell proliferation and somatic hypermutation to increase the affinity of their BCR for cognate antigen, followed by class switch recombination, accounting for different effector antibody functions (i.e., IgA, IgG, IgE) [35,42]. Hence—within GCs—a reservoir of memory B cells and long-lived plasma cells which secrete specific, high affinity antibodies, is generated.

It is important to note that T cell-dependent stimulation and antigen encounter may occur extra-follicularly, generating short-lived plasma cells instead [3], but this is beyond the scope of the current review article. In line with altered numbers of transitional B cells in AAV, an increased percentage of naive B cells was discovered in AAV patients regardless of disease activity [39] and in GPA patients in remission [33,38] compared to HCs. Interestingly, transitional and naive B cells in active GPA patients seem to be more responsive and sensitive to BCR stimulation [43], a characteristic normally attributed only to memory B cells (Bmem) [3].

Both MZB and follicular B cells can differentiate into non-switched (IgD^+^CD27^+^) or switched (IgD^−^CD27^+^) Bmem in a GC-dependent or -independent manner [44]. The fate decision seems to be dependent on the duration of the contact between the B cell and its cognate T cell: longer contact would favour GC reaction, whereas a shorter one would promote GC-independent memory formation [44]. Switched Bmem cells are the precursors of the antibody-producing plasmablasts/PCs, which makes them important in the pathogenesis of AAV. A lower proportion of Bmem cells in GPA patients in remission was reported [38], while others found reduced Bmem numbers in peripheral blood of active AAV patients [41,45]. Of note, in the latter case Bmem frequency was restored during remission [45]. Despite disease activity, the observed lower Bmem cells may be due to an increased differentiation into plasma cells, which are absent in blood, or their migration to inflammatory sites [38].

PCs (CD20^−^CD38^+^CD138^+^) are the most differentiated B lineage cells and specialized in antibody production (reviewed in [46]). PCs can be short lived and found in secondary lymphoid organs, or long lived and located at specialized niches in the BM. PCs come in different flavours as both CD19^−^ and CD19^+^ phenotypes have been described [47,48]. PCs have also been found in inflammatory lesions of GPA patients [49], suggesting that they play a role in the perpetuation of inflammation in AAV. Interestingly, the bone marrow of patients with autoimmune diseases often contains activated plasmablasts [50], which may also be the case in AAV, but this has not been formally investigated yet.

PCs in AAV are known to produce ANCAs, of which anti-PR3 and MPO are the most clinically relevant [51]. ANCAs contribute to the pathogenesis of the disease by triggering vascular inflammatory cascades upon recognition of mainly PR3 or MPO on neutrophils. The ANCA levels in patients have been described to correlate with disease activity [52] and are often higher in active disease when compared to remission phases. ANCAs can also be present in HCs at lower levels [53], which suggests that the pathogenicity of ANCAs may rely on epitope specificity [19]. For instance, only MPO-ANCAs targeting pathogenic epitopes presented a correlation with disease activity in ELISA epitope-specific assays [12]. Further (indirect) support for a potential pathogenic role of ANCAs arose from therapeutic targeting of B cells with RTX, which resulted in reduced ANCA titers and diminished disease activity [54,55], but it is not yet completely clear whether ANCA titers correlate with disease activity or not [9], and thus better biomarkers for AAV are needed. Immunoglobulin G subtype 4 (IgG4) was found in the serum and tissue of AAV patients, and sometimes IgG4-expressing plasma cells were also present [56,57]. A recent study investigating IgG4 as potential marker of disease activity in vasculitis [58] found that the IgG4:IgG RNA ratio was higher in the peripheral blood of active GPA patients when compared to patients in remission, which may make it a more sensitive marker than ANCA titers. However, ANCA-specificity may provide a better correlation with the serum cytokine profile of patients than with their clinical diagnosis [19,59], and could be a more specific trait to classify patients in terms of disease activity.

The aforementioned B cell types (transitional, naive/follicular, Bmem) are mostly found in the circulation or in secondary lymphoid organs. Interestingly, in agreement with the localisation of PCs at sites of inflammation [49], CD20^+^ B cells have also been found in-situ [49,60], which has been demonstrated to correlate with the occurrence of relapses in GPA after peripheral B cell depletion with RTX [60]. Of note, the CD20^+^ B cells found in granulomatous lesions express PR3 and exhibit features of Bmem. In 2017, researchers succeeded to identify and phenotype PR3-specific B cells in the circulation of patients and HCs [61], demonstrating that the most enriched B lineage cells expressing PR3 in AAV patients are switched Bmem cells and plasmablasts, suggesting that they are actively selected and escape peripheral tolerance checkpoints in patients. However, transitional B cells did not seem to be the main PR3-enriched population in AAV. Thus, further phenotyping of these cells which may also reside in LN or BM is required to elucidate potential alterations in specific B lineage subsets, including aberrations in signalling pathways that may contribute to development of autoimmunity.

Besides their antibody-dependent functions, B cells can also present antigen to T cells in GCs, thereby promoting cellular immune responses. Interestingly, tertiary lymphoid organs containing GCs (also called ectopic lymphoid structures) in which B cells function as antigen presenting cells were observed in renal biopsies [62].

B cells not only secrete pro-inflammatory cytokines, such as IL-6 and tumour necrosis factor (TNF)-α, but can also express the anti-inflammatory cytokine IL-10. B regulatory cells (Bregs) producing IL-10 are essential to maintain immune homeostasis. Therefore, any alteration in their number and/or function can lead to autoimmunity. Two Breg subsets have been defined: CD24^hi^CD38^hi^ and CD24^hi^CD27^hi^ cells, which share some phenotypical features with transitional B cells, including the high expression of CD38 and CD27. In the context of AAV IL-10-producing Bregs were found to be decreased in the peripheral blood of active GPA patients, whereas the opposite occurred after remission [63]. A different study discovered an association between reduced Breg numbers with decreased IL-10 production and higher T cell activation in vitro, which may favour the occurrence of relapses in AAV [45]. Furthermore, a subset of IL-10 producing Bregs expressing CD5 was reduced during active disease, and its increase during remission was associated with a decrease in ANCA titers [64]. This study further confirmed the lower numbers of CD24^hi^CD38^hi^ in active AAV and CD24^hi^ CD27^hi^ cells in both disease activity states. However, despite the numerical changes in these cells, IL-10 production in remission was not different from HCs [39]. All in all, the suppressive capacity of Bregs may be reduced in AAV, which may add to disease activity.

## 4. Key Features of B Lineage Cells in AAV

B lineage cells require various soluble factors and cell-cell contact interactions, which are recognized by specific receptors, for their proliferation, differentiation, survival, and function. These cell responses are dependent on downstream signalling pathways that convey these signals to the nucleus [65]. Recent studies showed that AAV B lineage cells have a dysregulation of various receptors and downstream effector molecules. Furthermore, serum concentration of ligands critical for B lineage cell responses are altered in AAV patients in comparison to HCs. These alterations may contribute to disease pathogenesis by enhanced differentiation towards plasmablasts and PCs, increased (auto)antibody and cytokine production, and promotion of antigen presentation to T cells. Thorough understanding of these and other B-cell intrinsic mechanisms induced via distinct signalling pathways in AVV is essential for the identification of critical targets in AAV pathology.

### 4.1. Receptors and Ligands

**Figure 2:**
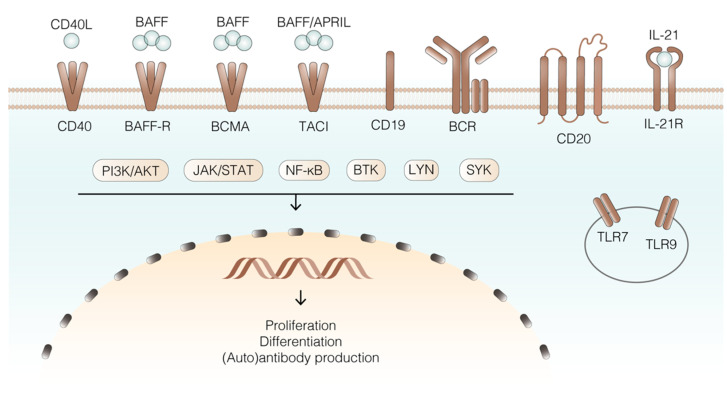
Selection of key receptors, ligands, signalling molecules, and transcriptional regulators of B lineage cells in AAV. B cells present many different receptors on their surface, each triggering specific signalling pathways that eventually lead to transcription of genes necessary for B cell specific and essential functions. Blue: ligands; brown: receptors; cream: effector molecules/signalling pathways. The circle represents the endosome, and the yellow semicircle the nucleus. Abbreviations: BAFF (B cell activating factor), APRIL (a proliferation-inducing ligand), BAFF-R (B cell activating factor receptor), BCMA (B cell maturation antigen), TACI (transmembrane activator and cyclophilin ligand interactor), BCR (B cell receptor), TLR (toll-like receptor), BTK (Bruton’s tyrosine kinase), SYK (spleen tyrosine kinase), NF-κB (nuclear factor kappa-light-chain-enhancer of activated B cells), JAK (Janus family tyrosine kinases), STAT (signal transducer and activator of transcription), PI3K (phpsphatidylinositol-3-kinase), LYN (Lck/Yes related novel protein tyrosine kinase).

Among the ligands that are critical for B cell responses are B cell activating factor (BAFF) and a proliferation-inducing ligand (APRIL) [66,67] (Figure 2), which belong to the tumour necrosis factor (TNF) superfamily (TNFSF). During B cell differentiation, different receptors from the TNFRSF are expressed, namely BAFF receptor (BAFF-R), transmembrane activator and cyclophilin ligand interactor (TACI) and B cell maturation antigen (BCMA), which have key roles in B cell differentiation and function [68]. BAFF is one of the two essential factors for proper B cell function, and after its processing, its soluble form may bind to BAFFR, TACI, or BCMA with specific outcomes [69]. BAFF is expressed by different cell types—including macrophages, neutrophils, dendritic cells, and monocytes [70,71,72,73]—and it was described that increased BAFF concentrations may lead to a less stringent B cell selection process, favoring survival of autoreactive clones [74]. Interestingly, BAFF levels were increased in active disease and even further increased after B-cell depletion with RTX, which may favour survival of autoreactive B lineage cells in the BM or facilitate relapse in some patients [70,73]. ANCAs themselves may be involved in BAFF induction as well, since total IgG obtained from AAV patients, but not control IgG, was capable of inducing BAFF production by neutrophils in vitro [73]. In patients, this could result in a vicious circle of B lineage cell activation. Despite the increased levels of BAFF in PR3-AAV [70] and MPO-AAV [65], few attempts have aimed to establish a relation between BAFF levels and disease activity. Initial studies on the potential correlation between BAFF levels and ANCA titers in MPO patients [65] led to the conclusion that this correlation existed only between BAFF levels and disease activity and not with MPO-ANCA levels [75]. In another GPA study, serum BAFF levels were higher in patients compared to HCs, but inversely correlated with ANCA titers [76]. In summary, the correlation between ANCA titre and BAFF levels remains unclear and the effect of BAFF on ANCA production still needs to be elucidated. Targeting soluble BAFF using the monoclonal antibody belimumab [77] resulted in beneficial effects in other autoimmune diseases, such as SLE [78,79], which suggest a potential specific targeting of only autoreactive B cells and IgG autoantibodies when compared to total IgG. Recently, a rationale was proposed for combination therapy of RTX and belimumab in AAV, which may result in more broad depletion of B cell lineage cells, including Bmem, both in the tissue and in the circulation (reviewed in [26]). Of note, a phase II study of rituximab and belimumab combination therapy in PR3 vasculitis is currently ongoing (COMBIVAS) [80].

BAFF-R is expressed early during B cell maturation, particularly on transitional and naive B cells [81,82], while its expression is lower in the GC. BAFF is the only ligand for BAFF-R [83], their interaction being crucial in the earliest transitional B stage (T1) [84]. BAFF-BAFF-R interaction triggers the activation of the nuclear factor kappa light chain enhancer of activated B cells (NF-κB) and mitogen-activated protein kinase (MAPK) pathways, promoting B cell survival [69,84]. BAFF-R expression on B cells in GPA was lower compared to HCs [85]. Furthermore, this study reported an inverse correlation of BAFF-R expression and circulating BAFF levels, which is consistent with previous data. This can be explained from previous observations in which BAFF-R was internalized upon ligation of ligands, BAFF levels also being negatively correlated with the expression of BAFF-R [86].

BAFF, as well as APRIL, can bind to TACI, which is expressed on some mature naive B cells, Bmem and plasmablasts [66], and to the receptor BCMA, which is mainly expressed on PCs (reviewed in [26]). Of note, BAFF and APRIL are present in high concentrations in serum and in tissue lesions of AAV patients [69,70,87], suggesting that BAFF and APRIL-induced B cell activation may take place both in target tissues and in periphery. However, no correlation between ANCA titers and APRIL was found in AAV [65].

BCMA is mainly expressed on plasmablasts and PCs, and has the highest affinity for APRIL, though it can also bind BAFF [69]. BCMA activates the MAPK pathway and enhances anti-apoptotic processes to promote cell survival [88]. Upon expression of BCMA, BAFF-R is downregulated [89], and BCMA is required to ensure long-lasting antibody production by PCs [90]. Of note, secreted APRIL was found to colocalize with the PC marker CD138 in inflamed nasal tissue of GPA patients, where a high expression of RANKL (receptor activator of NF-κB ligand) in PCs was detected. The findings of this study suggested that the increased RANKL expression may be indirect evidence of a potential role of BCMA upon APRIL ligation in GPA pathogenesis [87].

CD40 is another member of the TNF receptor superfamily [91] expressed on B cells and other antigen presenting cells. Signals downstream of CD40 are triggered by binding of CD40 ligand (CD40L), a trimeric protein expressed by T cells [92,93]. Among the functions downstream of CD40 signalling are B cell proliferation, GC formation and GC reactions, and Bmem differentiation upon T-cell dependent responses [94]. CD40 is important for NF-κB activation, and regulation of apoptosis [94,95], and its soluble form was found to be upregulated in AAV [96].

The BCR is central in the differentiation and effector function of B lineage cells. For instance, BCR triggering enhances BAFF-R signalling, which is necessary for a more mature transitional B cell population, named T2 cells. The importance of the BCR is further illustrated by the implication of various of its downstream BCR signalling molecules, including Lck/Yes related novel protein tyrosine kinase (LYN), spleen tyrosine kinase (SYK), Bruton’s tyrosine kinase (BTK) [97], and phosphatidylinositol-3 kinase (PI3K) [98] in antibody-mediated autoimmune diseases (reviewed in [99]). Interestingly, BCR signalling also contributes to the production of important effectors of the NF-κB signalling pathway [26,100]. The BCR has different co-receptors, including CD19 and CD20, which enhance its signalling capacity. CD19 has been found to be dysregulated both in SLE and AAV, distinguishing between low and high expression in different B cell populations [101]. Naive B cells were CD19^low^, leading to weaker downstream BCR signalling and probably the escape from selection mechanisms for self-recognition. However, Bmem were CD19^hi^, which makes them more sensitive to BCR signalling and therefore to proliferation and differentiation [101]. CD20 is a transmembrane protein on B cells [102] which was found to be involved in BCR and CD40 signalling. CD20 is expressed throughout all B cell development stages, but it is downregulated in PCs [103]. As mentioned, CD20 is one of the most relevant targets on B cells, as shown by the benefits of B cell depletion by the anti-CD20 antibody RTX.

IL-21 receptor (IL-21R) is part of the T cell dependent response [104] and its ligand, the cytokine IL-21, was first discovered to be produced by activated T cells [105]. IL-21R is expressed by naive and GC B cells [106], and its expression increases in Bmem and PCs during their differentiation. IL-21R is induced upon T cell dependent stimulation via CD40 and the BCR and its signalling contributes to B cell activation [104,106]. Hence, IL-21/IL-21R seem to have an important role in the B cell fate. Upon IL-21 stimulation, signalling pathways are triggered, including the Janus family tyrosine kinase (JAK)/signal transducer and activator of transcription (STAT), PI3K/AKT and MAPK (reviewed in [104]) pathways, eventually leading to key B cell functions—including proliferation, GC reactions, Ig production, and PC differentiation [107,108]. The JAK/STAT signalling pathway is mainly triggered by cytokine stimulation, and very recently targeting of this pathway in AAV resulted in promising results. This small study analysed the safety and efficacy of tofacitinib, a JAK1/3 inhibitor in a small cohort of GPA, MPA, and EGPA patients [109]. Even though further research in a larger clinical trial is needed, AAV patients presented improved clinical symptoms and inflammation markers, and tofacitinib showed both a good toleration profile and effectivity as a potential treatment for AAV patients.

Toll-like receptors (TLRs) have an important role in B cell activation and they are part of the innate immune response during infections [110], though an important role in autoimmune diseases has also been described [111]. For example, stimulation of TLR9 with its ligand CpG, which is present in bacterial DNA induces B cell activation in a T cell-independent manner [112]. It has been suggested that TLR9 and TLR7 may be implicated in autoimmunity, either by promoting IFN production or by activating self-reactive B cells. TLR9 may be an interesting target in AAV as CpG stimulation in combination with BAFF/IL-21 signalling had a synergistic effect on PR3-ANCA production in vitro [85]. In addition, in vitro stimulation of TLR9 with CpG increased the expression of TACI on naive B cells, decreased the percentage of TACI^+^ Bmem, and increased IL-21R on Bmem of GPA patients, which may favour their activation [85]. Furthermore, TLR9 stimulation with CpG induced a significant increase of PR3-ANCA production by B cells obtained from AAV patients [112]. Recently, it was shown that increased TLR9 expression in PCs upon stimulation with high mobility group box 1 (HMGB1) in MPO-AAV correlated with disease activity [113], suggesting—in line with the previous reports—that TLR9 expression could be pathogenic in AAV.

### 4.2. Intracellular Signalling Pathways

Downstream of the aforementioned receptors on B cells, several molecular effectors have been found to be dysregulated in antibody-mediated autoimmune diseases, revealing potential novel therapeutic targets that could be blocked with antibodies or small molecule inhibitors. LYN is a non-receptor tyrosine kinase belonging to the non-receptor Src-family kinases (SFKs) which can localize to the cell membrane and lipid rafts [114]. LYN can trigger several downstream effector molecules that promote B cell responses [115]. LYN on itself is activated through phosphorylation upon BCR signalling, leading to the activation of downstream effector molecules like SYK [116]. However, LYN can also negatively regulate B cell function. This negative loop is triggered via the phosphorylation of inhibitory receptors on B cells, including CD22 and FcγRIIB [117], which allows for the activation of important phosphatases that will downregulate signalling cascades mediated by phosphorylation loops [114]. Another mechanism to regulate BCR signalling involves LYN-mediated BCR internalisation [118]. B cell specific deletion of Lyn in mice leads to AKT hyperphosphorylation, resulting increased BCR sensitivity to stimulation and autoimmune features, such as autoantibody production and glomerulonephritis [119]. SLE patients also exhibited decreased LYN expression [120,121]. As SYK is altered in AAV (see section below), a dysregulation of its upstream effector LYN could account for this, but this has not been investigated yet.

SYK is an important effector downstream of early BCR signalling. This kinase initiates several signalling pathways: Ca^2+^ influx via nuclear factor of activated T cells (NFAT), BTK and PI3K signalling (both leading to the activation of NF-κB), and MAPK signalling. Fostamatinib, an oral SYK inhibitor, showed promising results at impairing B cell development, particularly at the transitional B cell stage [122], in patients with B cell lymphoid malignancies. Both BCR and BAFF-R receptor signalling promote the phosphorylation of SYK, which was found to be upregulated in active inflammatory glomerular lesions of AAV patients [123]. However, although this study focused on monocytes/macrophages, the results suggest that B cells also could over-express this protein. Upon BCR signalling, SYK phosphorylates CD19, which promotes the recruitment of PI3K to the membrane and subsequently results in activation of AKT [114,124,125,126,127] (Figure 2). AKT activation in turn leads to the phosphorylation of the transcription factor FOXO, preventing apoptosis [128] and promoting survival. AKT is also known to activate NF-κB signalling [129], thereby further promoting cell survival. PI3K promotes positive selection of B cells during their development, and once in the periphery, this kinase can be triggered in B lineage cells by the BCR, CD40, and TLRs [130,131,132,133].

BTK has a key role in BCR-mediated signalling [134,135]. A recent study focused on BTK activity in two B cell subsets in GPA patients: transitional and naive B cells [43]. Interestingly, BTK levels were found to be increased in both subsets in active disease when compared to remission patients and HCs. Furthermore, targeting of BTK in these cells inhibited B cell cytokine production, differentiation, and (auto)antibody production, the effects being more significant in remission. Despite the lack of reports on dysregulated PI3K signalling in AAV, the increased BTK levels in AAV B cells could provide indirect evidence of aberrations in upstream kinases.

NF-κB signalling is essential for many B cell functions, including B cell proliferation, differentiation, and antibody production [136], and it has been shown to be involved in many B cell mediated diseases including B cell lymphomas [137] and various autoimmune diseases (reviewed in [138]). NF-κB not only has an important role in early B cell development, presumably via BAFF-R signalling [136], but also in Bmem cells for their survival and antibody production. Hence, dysregulations of NF-κB signalling may favour maintenance of autoreactive cells. NF-κB can be activated via two interconnected pathways: the canonical pathway, which can be activated for instance via wide range of receptors expressed by B lineage cells—e.g., BCR, TLRs, CD40, BAFF-R, and the non-canonical pathway—which can be activated upon triggering of only a specific subset of TNFRSF members, such as CD40 and BAFF-R [139,140]. Of note, the two NF-κB pathways differ in their activation kinetics: the canonical pathway governs a quick but time-limited activation, whereas the non-canonical pathway needs to be induced, and induces a slow activation that is maintained in time [141]. The activation of these pathways converges in the transcription of specific genes in the nucleus. Key regulators of both pathways are the IκB kinase complex (IKK) consisting of three proteins (IKKα, IKKβ, and IKKγ) for the canonical, and NF-κB-inducing kinase (NIK) for the non-canonical pathway, the latter having been successfully targeted in SLE mouse models [142]. These findings, together with the relevance of NF-κB for B cells, make it an interesting potential novel therapeutic target in AAV.

### 4.3. Cytokines and Chemokines

B cells require various cytokines produced by other cell types for survival and proliferation, among other functions. Dysregulation at certain levels in this process may promote autoimmunity, as already discussed for the cytokines BAFF and APRIL. A recent study examined the association of cytokine profiles and ANCA-specificity in AAV and found that the different cytokine levels could better distinguish patients with AAV according to their ANCA specificity than to their clinical diagnosis [59]. Among the increased cytokines in PR3-AAV compared to controls were IL-6, granulocyte-macrophage colony-stimulating factor (GM-CSF), sIL-2Ra, IL-15, and IL-18, whereas in MPO-AAV serum levels of sIL6R and sTNFRII were increased, which is in agreement with previous findings [143]. These cytokines contribute to the activation to the aforementioned signal transduction pathways in B cells and could thus, in part, account for the dysregulated intracellular signalling that is observed in AAV [59].

Besides alterations in cytokine levels, also several chemokines are upregulated in both PR3-AAV and MPO-AAV, which could thus potentially be specific biomarkers for this disease. As an example, CXCL13 or B cell attracting chemokine-1 (BCA-1) levels were higher in the serum of patients with active AAV, which may consequently be a potential biomarker to distinguish active from remission AAV [143]. Thymus-and-activation-regulated chemokine (TARC) or CCL17 was also higher in AAV, however, it was not considered to be a potential disease activity marker.

Not only cytokines affecting B cells are upregulated in AAV, but also cytokines produced by autoreactive B cells. As mentioned previously, the pathogenic role of B cells in AAV involves antibody-independent mechanisms, including inflammatory cytokine secretion and antigen presentation. B cells can secrete IL-6 and TNF-α upon CD40 [144], TLR [27], and IL-21R induced signalling events. IL-6 and TNF-α were present at higher levels in GPA and MPA patients when compared to HCs [145]. These cytokines contribute to maintenance of inflammation and B cell responses, for instance via inducing the differentiation of effector T cells [146]. However, more research is required to confirm that these cytokines are produced by B cells in AAV [27]. A-disintegrin and metalloproteinase domain-containing protein 17 (ADAM17) is responsible for ectodomain shedding of TNF-α, among other substrates, and its concentration was higher in plasma samples from active PR3-AAV when compared to remission state and HCs [147]. Interestingly, higher serum levels of IL-10 were found in AAV patients, which may be indicative of immunomodulatory mechanisms including Breg function in active disease. However, data in the current literature are conflicting, as other studies report lower Breg numbers in active disease that returned to normal levels during remission [63], whereas others observed similar numbers in active disease and remission [39].

## 5. Concluding Remarks and Future Perspectives

AAV is a complex autoimmune disease in which existing research suggests a crucial role of B cells and plasma cells in its pathogenesis. Moreover, B lineage cells not only have autoreactive antibody-dependent functions, but they can also act as antigen presenting cells and secrete inflammatory cytokines. Although different therapeutic strategies to target these cells—including the widely used B cell depleting agent RTX—are available to treat AAV patients, there is still a need for additional therapies targeting B lineage cells. Advanced understanding of the (intrinsic) molecular mechanisms driving autoreactive B cell proliferation, differentiation, and autoantibody production in AAV may enable a more targeted approach.

This review gathers evidence of dysregulated B cell lineage populations in AAV, which suggests an essential role of some of them in the pathogenesis of this disease. Importantly, research in AAV is now moving in the direction of finding more targeted therapies to fill the existing gap, as is clear from more recent studies on B cell signalling and its targeting in AAV that were discussed in this review. From these, some important receptors have already been targeted, as well as some of their downstream effector molecules, including BTK and SYK with promising results. B cell cytokines have also been subject of therapeutic targeting, such as the BAFF-targeting monoclonal antibody belimumab, yet deeper knowledge on this subject and formal clinical trials are required. It is essential to remember that even though it has been an important step in research in AAV, studies focused on B cell signalling were mainly performed in B cells from peripheral blood, not knowing to what extent aberrantly regulated pathways and potential therapeutic targets would also be operational in plasmablasts and PCs, which predominantly reside in lymph nodes and specific niches in the BM. Hence, it is crucial to advance our understanding of plasmablasts and PCs by performing lymph node and BM analysis in AAV to achieve the unmet clinical needs of specifically targeting these cell types in AAV. For instance, PCs could probably be better targeted using anti-CD38 therapies or perhaps even CD19-directed CAR-T cell therapy, as was found to be promising in SLE [148,149]. Currently, the only therapeutic proven to be effective targeting PCs in AAV is bortezomib, a proteasome inhibitor which efficiently depleted PCs in a mouse model of AAV [150], resulting in lower anti-MPO titers and prevention of glomerulonephritis. Interestingly, bortezomib has also been effectively applied and induced long-term remission in a therapy-refractory AAV patient [151]. However, this treatment has considerable side-effects and the effects of bortezomib on PCs were not evaluated in the human studies.

All in all, the current literature and ongoing research projects provide evidence that unravelling the (intrinsic) signalling mechanisms in B cell lineage cells that contribute to pathology may yield new potential therapeutic targets that could be exploited for specific intervention in or depletion of activated and pathogenic B cell populations including PCs, preferably without interfering with other functions of the immune system.
